# All-Cause Mortality and Life Expectancy by Birth Cohort Across US States

**DOI:** 10.1001/jamanetworkopen.2025.7695

**Published:** 2025-04-28

**Authors:** Theodore R. Holford, Lisa McKay, Jamie Tam, Jihyoun Jeon, Rafael Meza

**Affiliations:** 1 Department of Biostatistics, Yale School of Public Health, New Haven, Connecticut; 2 Currently retired; 3 Department of Health Policy and Management, Yale School of Public Health, New Haven, Connecticut; 4 Department of Epidemiology, School of Public Health, University of Michigan, Ann Arbor; 5 Department of Integrative Oncology, British Columbia Cancer Research Institute, Vancouver, Canada; 6 School of Population and Public Health, University of British Columbia, Vancouver, Canada

## Abstract

**Importance:**

Although overall US mortality rates declined from 1969 to 2020, they vary considerably by state and generation, especially when evaluated by birth cohort. Trends in mortality and life expectancy by birth cohort for US states and Washington, DC, have yet to be characterized.

**Objective:**

To estimate cohort mortality trends for each state and Washington, DC, and quantify life expectancy at birth and 40 years of age and the rate of increase after 35 years of age.

**Design, Setting, and Participants:**

In this cohort study, all-cause mortality rates by single years of age (0-119) and birth cohort (1900-2000) were estimated for each state in January 2025. Mortality data and population estimates were obtained from the National Center for Health Statistics, the Centers for Disease Control and Prevention Wide-Ranging Online Data for Epidemiologic Research website, and the Surveillance, Epidemiology, and End Results database for each state and Washington, DC, by single years of ages 0 to 84 and calendar years 1969 to 2020. An age-period-cohort model with constrained cubic splines for temporal effect estimates was used to estimate mortality from 1900 to 2000.

**Main Outcomes and Measures:**

Life expectancy for each cohort from birth or 40 years of age was estimated by sex and state, along with doubling time for the death rate after 35 years of age.

**Results:**

Analyses included 179 million deaths (77 million female and 102 million male). In the West and Northeast, cohort life expectancy improved from 1900 to 2000, but in some Southern states, it changed less than 3 years since 1900 in females and less than 2 years since 1950 in males. Washington, DC, had the lowest life expectancy in the 1900 birth cohort but a greater increase than the other states (from 61.1 to 72.8 years of age). After 35 years of age, the highest rate-doubling time in a state was 9.39 years in New York for females and 11.47 years for males in Florida. The shortest rate-doubling times were 7.96 years for females in Oklahoma and 8.95 years for males in Iowa.

**Conclusions and Relevance:**

Cohort-specific patterns across states reveal wide disparities in mortality. Some states have experienced little or no improvements in life expectancy from the 1900 to 2000 birth cohorts. Understanding how mortality patterns vary by birth cohort within each state can inform decision-making around resource allocation and public health interventions.

## Key Points

**Question:**

How did cohort life expectancies change by US state during the 20th century?

**Findings:**

In this cohort study of 179 million deaths, cohort life expectancy in the South, especially among females, changed little for those born from 1900 to 2000. For several states in the West and Northeast, cohort life expectancy improved substantially, and Washington, DC, exhibited the greatest improvement.

**Meaning:**

These findings suggest that understanding how mortality patterns vary by birth cohort within each state can inform decision-making around resource allocation and public health interventions.

## Teaser

This cohort study characterizes trends in mortality and life expectancy by birth cohort for US states and Washington, DC.

## Introduction

A primary focus of the United Nations Sustainable Development Goals is the reduction of premature mortality in every country.^1^ Prior to some recent declines in US life expectancy,^2^ the US had experienced a considerable decrease in mortality rates and increasing life expectancy attributed to improvements in sanitation, tobacco control, health care, and prevention of cardiovascular disease, cancer, and other diseases.^3^ While overall mortality trends for the US have improved throughout the 20th century, there is considerable geographic variability by state. An analysis of life expectancy from 1959 to 2017 found differences widening between states, particularly after the 1980s and 1990s.^4^ These are undoubtedly shaped by state-level policies: states with progressive public health policies were more likely to experience increases in life expectancy than states without such policies.^5^ Spatial differences in mortality rates are also observed even at more granular levels by county.^6,7^

In the US, health policy is often established and implemented by states, and for many years, the effect of socioeconomic differences among the states has resulted in substantial differences in the health environment. This study reports on trends in mortality in each state that can help to understand the setting for health policies. Available data on state differences in mortality are usually analyzed cross-sectionally and not by birth cohort. While cross-sectional data allow for trend analysis by age group,^8^ each year includes different birth cohorts. Following cross-sections shifts the mixture of birth cohorts, so the generations are changing.^9^ For example, if following trends for people at 40 years of age, a 40-year-old individual born in 1950 would have different health experiences and mortality risks than one born in 1980. Herein we describe the differences in the period and cohort perspectives to better understand ways in which medical and public health policies can impact the health of different generations. The experience of a cohort early in life can shape exposures and risk factors that are carried forward as the group ages.^10,11,12^ Public health interventions often affect a cohort earlier in life, and the impact then follows them as they get older, as illustrated by a recent study of the potential impact of changing the minimum age for legal purchase of cigarettes.^9^ Summarizing mortality trends by birth cohort is thus more reflective of lived experiences of populations. This highlights the importance of the birth cohort perspective, a perspective that is largely absent from the usual summaries of state mortality data. This study aims to fill this gap by characterizing trends in mortality and life expectancy by birth cohort for US states and Washington, DC.

## Methods

### Data Sources

This study was approved by the Institutional Review Board of Yale University School of Medicine. This study used publicly available data collected by agencies of the US government, and no further contact with individuals or informed consent was required. The study complies with Strengthening the Reporting of Observational Studies in Epidemiology (STROBE) reporting guideline for cohort studies. In this cohort study, all-cause mortality rates by single years of age (0-119) and birth cohort (1900-2000) were estimated for each state in January 2025. Data on all-cause mortality for each state and Washington, DC, were obtained by single years of age (0-84) and calendar year (1969-2020) from the Centers for Disease Control and Prevention (CDC) National Center for Health Statistics (NCHS), as shown in eFigure 1 in Supplement 1. For ages 1 to 84 years, the number of deaths for each year is available online for years 1969 to 1998.^13^ However, in subsequent years, online files suppress reporting of frequencies less than 10, resulting in biased estimates if these observations are dropped. For years 1999 to 2020, unrestricted vital statistics NCHS data were obtained with permission from NCHS.^14^ The number of deaths at younger than 1 year used the number of infant deaths reported by the CDC Wide-Ranging Online Data for Epidemiologic Research database.^15,16,17^ These files also do not report frequencies less than 10, so in some instances, sex- and state-specific frequencies were estimated by taking the difference between the total number of deaths and the number for the other sex, thus completing the data file. Population estimates were obtained from the Surveillance, Epidemiology, and End Results Program database.^18^

### Statistical Analysis

An age-period-cohort model was used to analyze temporal trends. Birth cohorts represented in the data begin in 1885 for individuals who were 84 years of age in 1969 through 2020 for those more recently born. For each state, a log-linear model was fitted to the mortality rate at age *x* and cohort *t*, similar to the equation of Lee and Hsieh,^19^

ln*m*(*x*,*t*) = *μ* + *α*(*x*) + *γ*(*t*) + *π*(*t* + *x*)

where μ is the intercept; *α*(*x*), the estimated effect of age *x*; *γ*(*t*), the estimated effect of cohort *t*; and π(*t* + *x*), the estimated effect of period. The number of deaths is assumed to have a distribution in which the variance is proportional to the mean, fitted using a generalized linear model, PROC GENMOD in SAS statistical software, version 9.4 (SAS Institute Inc). Estimated temporal effects were represented by constrained cubic splines in which the trends are constrained to be linear before the first and after the last knot.^20^ For age, a dummy variable was given for infants, 0 years of age, and knots were set at 2, 5, 10, 15, 25, and 35 years of age, which forces the estimated effect to be linear for 1 to 2 years of age or older than 35 years. The linear trend for the log rate after 35 years of age is the association specified by the Gompertz model.^21^ This analysis focuses on trends since birth and adults 40 years or older. For the cohort estimated effect, knots were placed in 1900, 1910, 1920, 1930, 1940, 1950, 1955, 1960, 1965, 1970, 1975, 1980, 1985, 1990, 2000, and 2010. The period slope is set to zero, and knots are set at 1975, 1980, 1985, 1990, 1995, 2000, 2005, 2010, and 2015. Mortality in 2020 began to experience a surge caused by COVID-19, but the splines used to estimate mortality rates forced the period effect estimate to be linear after the last knot, 2015. This would temper the effect of the surge on these estimates and offer a better summary of long-term trends in mortality. Results from fitting this model provided mortality rate estimates for single years of age (0-119) and cohort (1885-2020) for each state. The rate estimates were constrained to be less than 1.

Both period and cohort life expectancies were calculated. Period life expectancy, the more commonly used method, uses the death rates for a given year and determines the life expectancy for a hypothetical population experiencing those rates.^22^ This was calculated from birth until 119 years of age for calendar years 1969 to 2020. For cohort life expectancies, the model estimates death rates for each age, 0 to 119 years, for cohorts born from 1900 to 2000. These rates were used to estimate the expected number of remaining years of life at any given age, which were reported from birth and from 40 years of age (eFigures 4-7 in Supplement 1) for each period and birth cohort.

The model used sets a linear trend for after 35 years of age with the slope β, which measures the rate at which the death rate increases with age.^21^ To more easily interpret this trend, time to double the mortality rate after 35 years of age is used, (log 2)/β. A steeper increase in the mortality rate yields shorter doubling times, which are shown for each state. Two-sided *P* < .05 indicated statistical significance. Two-sided 95% confidence limits are shown in eTables 1 and 2 in Supplement 1.

Neither race nor ethnicity is reported herein to maximize precision for estimates in all states. We report biological factors and refer to sex. No patients were generated from this work and permission to use these data was obtained from the National Center for Health Statistics.

## Results

Analyses used 179 million deaths (77 million female and 102 million male) in the US. Model estimates of death rates showed strong agreement with observed estimates in each state, as reported for California in eFigure 2 in Supplement 1. A comparison of typical differences in trend among states is demonstrated for Washington, DC, New York, and Oklahoma in eFigure 3 in Supplement 1.

**Figure 1.**
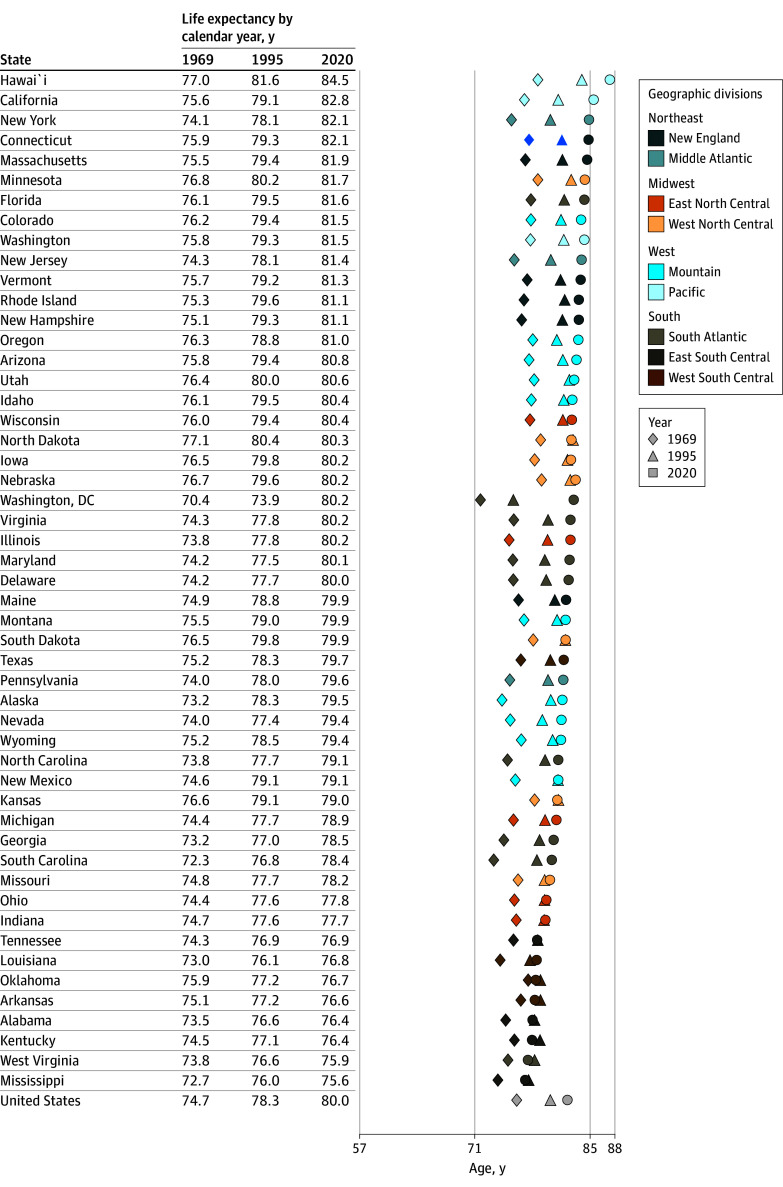
Period Life Expectancy at Birth for US Females by State and Calendar Year (1969, 1995, and 2020) Geographic divisions and regional distinctions are based on Centers for Disease Control and Prevention classifications.^23^

**Figure 2.**
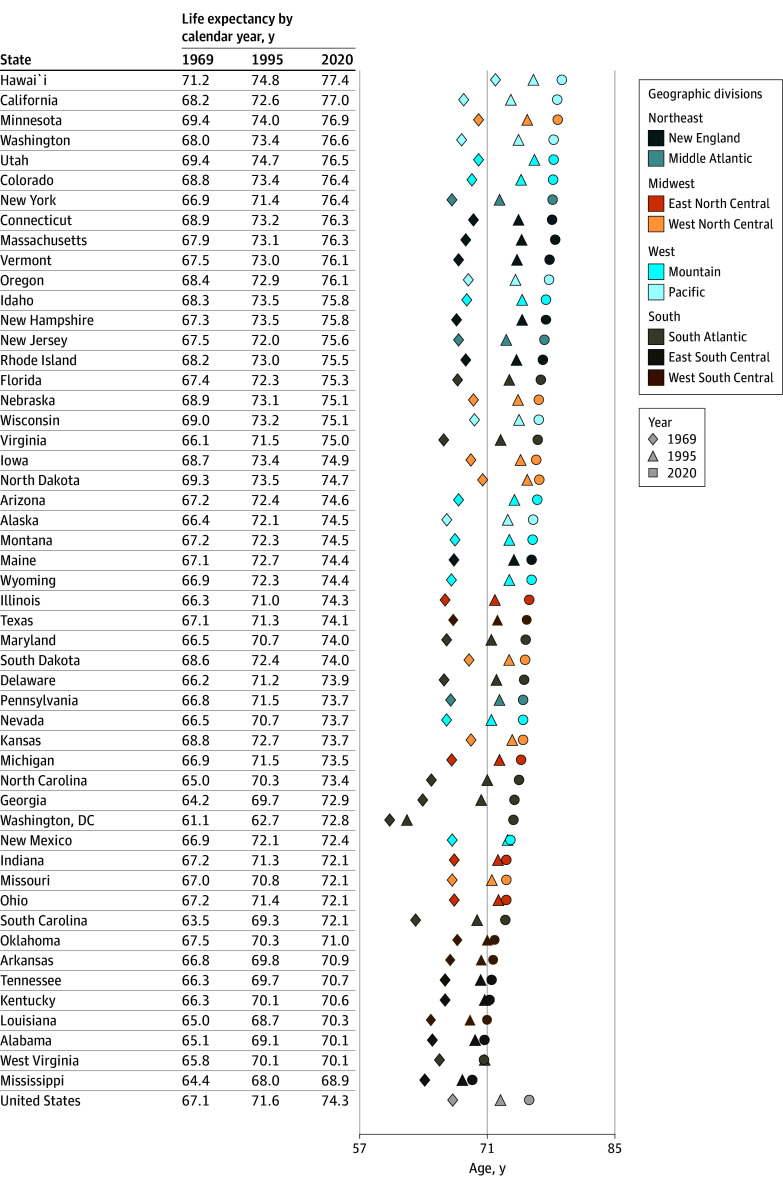
Period Life Expectancy at Birth for US Males by State and Calendar Year (1969, 1995, and 2020) Geographic divisions and regional distinctions are based on Centers for Disease Control and Prevention classifications.^23^

Figure 1 and Figure 2 show the period life expectancy at birth in each state for years 1969, 1995, and 2020, ranked from highest to lowest in 2020. For females, states in the West and Northeast tended to have the highest life expectancy and states in the South the lowest (Figure 1). Hawaii had the highest life expectancy (84.5 years in 2020), and Arkansas (76.6 years in 2020), Alabama (76.4 years in 2020), Kentucky (76.4 years in 2020), West Virginia (75.9 years in 2020), and Mississippi (75.6 years in 2020) had the lowest. States with the lowest life expectancy also showed less change than other states, especially after 1995. Washington, DC, had the greatest change, increasing life expectancy from 70.4 to 80.2 years. Males showed an increase in life expectancy for each state, although the change was smaller after 1995 for states with the lowest life expectancy in 2020: Arkansas (69.8 to 70.9 years), Tennessee (69.7 to 70.7 years), Kentucky (70.1 to 70.6 years), Louisiana (68.7 to 70.3 years), Alabama (69.1 to 70.1 years), West Virginia (70.1 to 70.1 years), and Mississippi (68.0 to 68.9 years) (Figure 2). Washington, DC, showed an even greater increase in life expectancy from 62.7 to 72.8 years of age. Rankings differed somewhat by sex, though the same states tended to appear in the lowest ranking. Similar results for life expectancy at 40 years of age are shown in eFigures 4 and 5 in Supplement 1.

**Figure 3.**
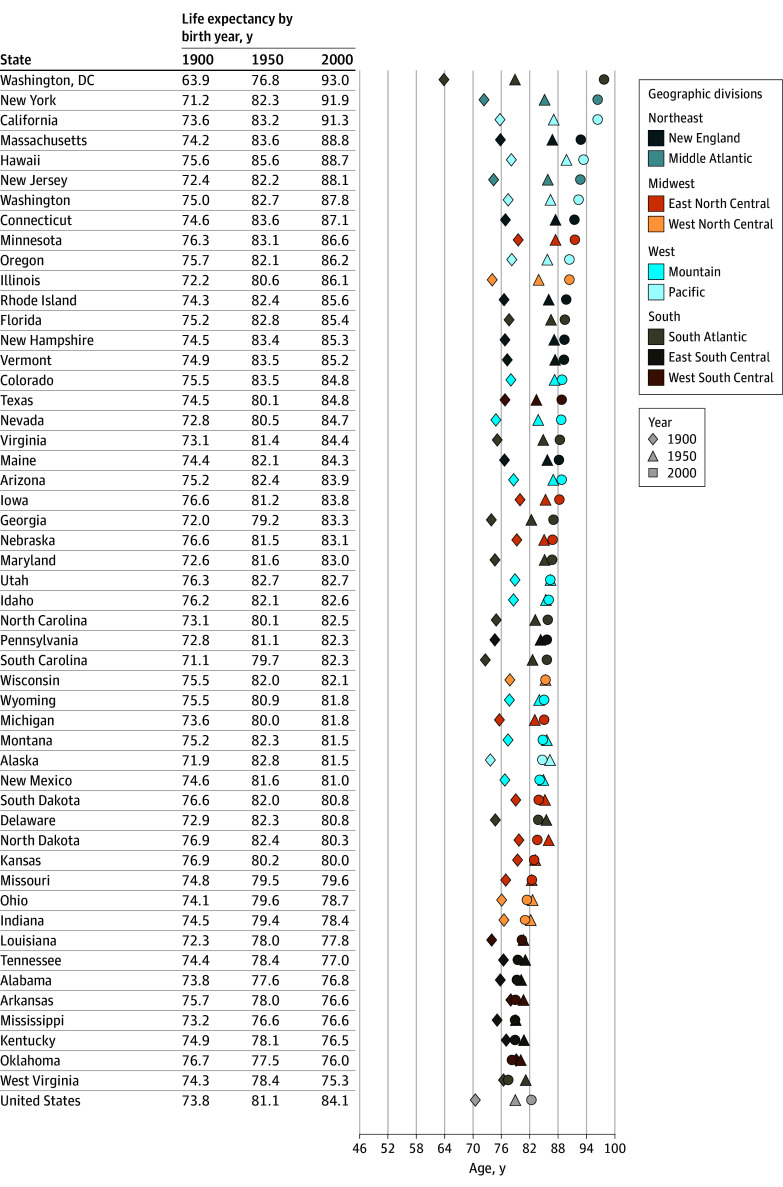
Cohort Life Expectancy at Birth for US Females by State and Birth Year (1900, 1950, and 2000) Geographic divisions and regional distinctions are based on Centers for Disease Control and Prevention classifications.^23^

**Figure 4.**
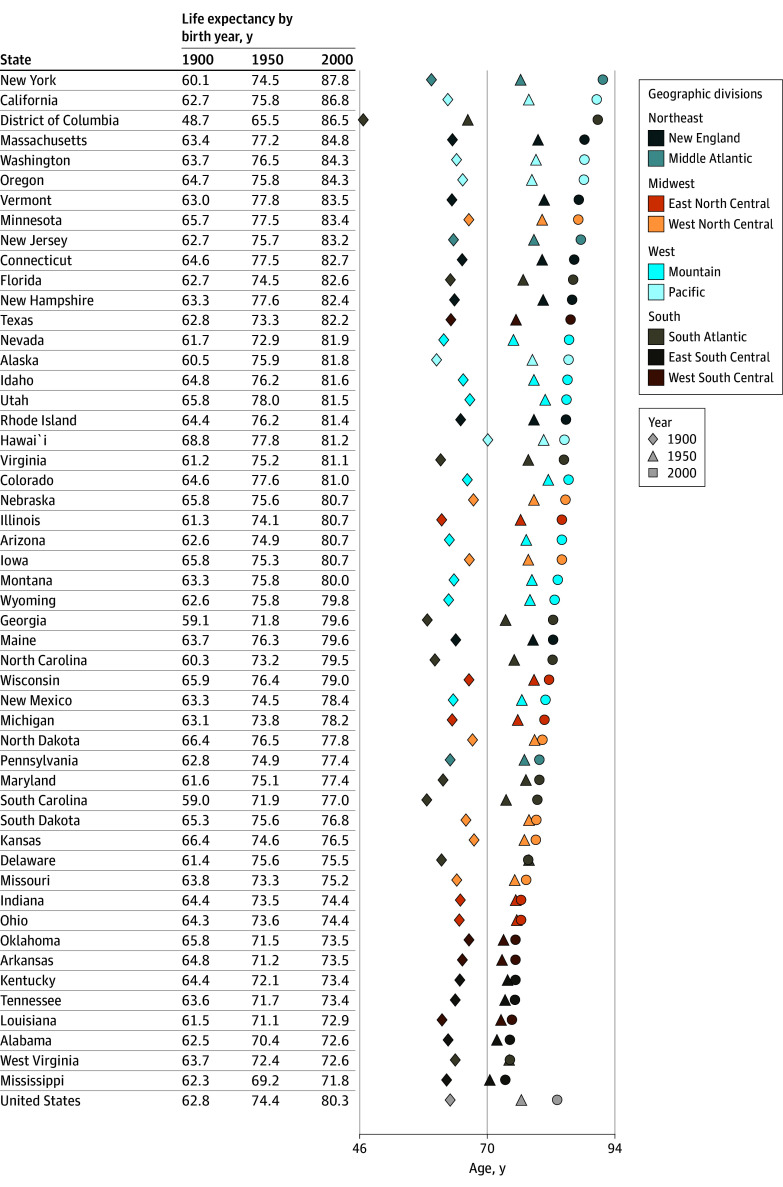
Cohort Life Expectancy at Birth for US Males by State and Birth Year (1900, 1950, and 2000) Geographic divisions and regional distinctions are based on Centers for Disease Control and Prevention classifications.^23^

Figure 3 and Figure 4 show cohort life expectancy at birth by state and cohort (1900, 1950, and 2000), ordered from highest to lowest in 2000. Life expectancies were generally higher for females than males and increased with cohort. However, in many Southern states, there was little change from 1900 to 2000 in females, but higher life expectancies and greater improvement occurred in the Pacific and Northeast (Figure 3). For many of these states, males showed some improvement up to 1950, which decreased to less than 2 years thereafter (Figure 4). The greatest observed change was for Washington, DC, which had the lowest cohort life expectancies in the 1900 cohort (63.9 years for females and 48.7 years for males) and the highest in 2000 for females (93.0 years) and the third highest for males (86.5 years). Life expectancies in 2000 were only slightly different than that of Washington, DC, in New York (91.9 years for females and 87.8 years for males) and California (91.3 years for females and 86.8 years for males). For both sexes, the 8 states with the lowest life expectancy are in the South (Oklahoma, Arkansas, Kentucky, Tennessee, Louisiana, Alabama, West Virginia, and Mississippi). Life expectancy in some of these states changed less than 3 years from 1900 to 2000 in females and less than 2 years from 1950 to 2000 in males. Life expectancies at 40 years of age by cohort are shown in eFigures 6 and 7 in Supplement 1.

The doubling times after 35 years of age, showing the rate of increase in mortality, a measure of the rate that a cohort is aging, are shown in the Table. These are shown with confidence limits in eTables 1 and 2 in Supplement 1. In each state, the mortality doubling time is higher for females than for males, although there is considerable variation by state. Washington, DC, is an outlier with doubling times of 12.27 for females and 15.64 years for males. Other states had rate doubling times of 7.96 to 9.39 for females and 8.95 to 11.47 for males. Pearson correlation between the doubling times for males and females in each state was 0.96 overall, but only slightly less, 0.88, if Washington, DC, was dropped. Doubling times were also correlated with life expectancy in 2000 (*r* = 0.49 in females [*P* < .001] and *r* = 0.28 in males [*P* = .046]). States in the Midwest tend to have faster doubling times for both females and males. For females, Oklahoma has the shortest doubling time, and those in Iowa and Kansas were also quite short. New York and Florida had relatively higher doubling times. Estimated age-specific mortality rates by state, sex, and cohort, along with additional figures showing life expectancy, are available on a data repository website.^24^

**Table.** Years Needed to Double the Death Rate After 35 Years of Age by Sex and State in the US^a^

State	Rate-doubling time, y	
	Females	Males
Alabama	8.50	10.13
Alaska	9.07	11.02
Arizona	8.94	11.02
Arkansas	8.17	9.75
California	9.05	10.52
Colorado	8.44	9.90
Connecticut	8.59	9.77
Delaware	8.92	10.37
Florida	9.28	11.47
Georgia	9.02	10.74
Hawaii	9.15	9.84
Idaho	8.05	9.38
Illinois	8.80	10.15
Indiana	8.23	9.30
Iowa	7.98	8.95
Kansas	7.98	9.09
Kentucky	8.18	9.62
Louisiana	8.92	10.55
Maine	8.22	9.51
Maryland	8.87	10.34
Massachusetts	8.60	9.90
Michigan	8.63	9.83
Minnesota	8.17	9.22
Missouri	8.39	9.76
Mississippi	8.68	10.18
Montana	8.50	9.89
Nebraska	8.14	9.15
Nevada	8.93	10.46
New Hampshire	8.28	9.40
New Jersey	8.82	10.08
New Mexico	8.87	10.97
New York	9.39	11.07
North Carolina	8.74	10.62
North Dakota	8.29	9.26
Ohio	8.39	9.42
Oklahoma	7.96	9.39
Oregon	8.29	9.60
Pennsylvania	8.60	9.89
Rhode Island	8.52	9.61
South Carolina	9.25	11.06
South Dakota	8.46	9.58
Tennessee	8.39	9.94
Texas	8.53	10.17
Utah	8.05	9.67
Vermont	8.24	9.70
Virgina	8.62	10.06
Washington	8.45	9.64
Washington, DC	12.27	15.64
West Virginia	8.28	9.83
Wisconsin	8.19	9.13
Wyoming	8.25	10.03
United States overall	8.72	10.18

^a^ Includes those who died from 1969 to 2020 by state.

## Discussion

This cohort study is the first, to our knowledge, to provide a comprehensive history of mortality trends across US states by birth cohort. We improve on existing state-specific mortality data that have focused primarily on trends by period (calendar year). The Gompertz model^21^ for cohort mortality trend after 35 years of age aligns well with the mortality data for each state and Washington, DC. Although some cohort-specific trends can be gleaned from observational data, the fitted models can be used to extrapolate mortality beyond the range of age and cohort where data are available, providing estimates for 85 years and older and cohorts born early in the 20th century. This reveals mortality changes across the life course between different generations across US states. We use a model for the estimated age effect that works well for the available data, but these estimates extend to 2119 for the 2000 cohort. One should use caution if relying on these estimates for prediction purposes, but these data provide a useful starting point for estimating the expected potential effects of health intervention strategies affecting cohorts. Nevertheless, uncertainty exists for these estimates, both from random error and future events and changes to health care. The rate of increase in adult mortality rate is characterized by the time to double the mortality rate after 35 years of age. Longer time for mortality to double suggests slower rates of increase in the death rate for a population. Mortality doubling time is consistently higher for males, indicating that their rate of increase in mortality with age is slower than that for females. However, the mortality rate itself remains higher for males than females over the entire life span. This pattern of lower but more rapidly increasing mortality by age in females vs males has also been found in comparisons of mortality by birth cohort and gender between different countries.^11^ The mortality rate doubling times vary considerably across states, with those for Washington, DC, being considerably higher than for the other states.

Variation in change in cohort life expectancy is large; for example, female cohort life expectancy in New York increased 20 years from 1900 to 2000 cohorts, but Oklahoma decreased by 0.7 years. Reasons for variation in the cohort life expectancies can be complex and include health behaviors such as cigarette smoking, drug use, environmental safety, social interactions, vaccination, and access to effective health care. In addition, infection and other causes of inflammation have been suggested as reasons for mortality trends.^12^ These can have differential effects on specific causes of death that affect the overall burden of disease in a state.^25,26^ Prior studies have considered mortality and disease from the perspective of calendar year or period but have not provided summaries for individuals by birth cohorts. The cohort trends can assess the outcomes of policy changes that have more impact on individuals of a particular age and impact health thereafter. Spatial trends can also occur at a finer geographic level, such as counties or towns, due to more localized factors affecting health.^6,7^ However, for small regions, the population size can make it difficult to estimate cohort life expectancy.

Persistent disparities in mortality rates reflect important state and regional differences in public policy, sociodemographic composition, rurality, and other social and behavioral norms that influence mortality outcomes.^5^ California was an early adopter of tobacco control policies, which influenced smoking and subsequent mortality trends. California implemented smoke-free workplace policies in 1995,^27^ which changed smoking behaviors and social norms for entire cohorts of young people born in the 1980s and 1990s who grew up surrounded by smoke-free environments, as well as generations of working-age adults who were prompted to quit. In contrast, Kentucky had essentially no such efforts to control cigarette smoking, resulting in higher cigarette use^28^ and, therefore, higher mortality compared with California. Similar patterns for smoking and mortality observed in Kentucky can be seen in West Virginia, Oklahoma, Arkansas, Tennessee, Louisiana, Mississippi, and Alabama. Lower socioeconomic status is associated with higher mortality risk; states that are socioeconomically advantaged in their demographic composition also appear to have improved mortality rates more quickly than other states. Many factors affect mortality, including policies related to labor, immigration, civil rights, and the environment.^5^

Washington, DC, has shown much greater change among cohorts born in the 20th century than the states, as seen in mortality rates, life expectancy, and time to double mortality after 35 years of age. However, unlike the 50 states, it is essentially an urban area, which often has better access to health care deand longer life expectancy than rural areas.^29^ In addition, its small size can make it sensitive to changes in the demographic mix of the population. Washington, DC, has a relatively small population compared with most states, so a relatively small number of migrating individuals may result in a relatively large change in its demographic distribution. This could reflect a pattern of gentrification as more affluent individuals, with better health care options, move into the nation’s capital.

### Strengths and Limitations

This study is strengthened by detailed comprehensive historical mortality data for each state and application of a linear model between log-mortality and temporal factors. The analysis makes use of the age-period-cohort model to generate these rates, which agrees well with available data, but it has limitations that are well recognized.^30^ Although demographic changes and migration patterns by state are not explicitly accounted for in the analysis, the underlying data reflect those shifts in the population composition for each state. The results reveal solid agreement between model estimates and observed mortality rates where data are currently available. However, this does not ensure that this agreement would have held in earlier years (pre-1969) or will continue in future years (post-2020). Geographic disparities in COVID-19–related mortality and the policies that shaped them after 2020 were not considered. Future research could examine how state-specific migration patterns, demographic composition, and COVID-19–related experiences shape mortality trends for future cohorts.

## Conclusions

In this cohort study of mortality by state in the US, both period and cohort life expectancy were estimated for males and females using an age-period-cohort model. From 1969 to 2020, period life expectancy increased, although there was greater improvement for some states in the West and Northeast and less for some states in the South. Some states in the West and Northeast showed increases in the cohort life expectancy greater than 30 years for those born in 2000 compared with 1900. However, in parts of the South, female cohort life expectancy increased by less than 2 years. For male cohorts in parts of the South, life expectancy increased from 1900 to 1950 but by less than 2 years after 1950.

State- or local-level decision-makers benefit from the availability of detailed mortality data that more accurately reflect their jurisdiction’s population. The birth cohort perspective is particularly important for capturing potential changes in mortality that would otherwise be masked in cross-sectional analyses. Understanding how these mortality patterns vary by birth cohort within each state can inform decision-making around resource allocation and public health interventions, especially those that may differentially affect birth cohorts. For example, policies directed at screening for disease or access to exposure affecting health are often directed at age groups, and the effect of these policies then follows cohorts as they get older. States with more progressive public health policies have generally fared better,^5^ and these are often more closely linked to cohort parameters. These mortality estimates can furthermore be used in simulation models to make projections about mortality trends in the future and how public policy intervention could alter those trajectories. Importantly, such data can facilitate surveillance of trends to determine whether state disparities are improving or worsening with more recent birth cohorts. If addressing geographic health disparities is indeed a nationwide priority, then this study contributes to the necessary data infrastructure for health equity progress.

## Notes

Supplement 1. **eMethods.** Data Collection and Analysis **eTable 1.** Years to Double the Death Rate After Age 40 by State and Cohort in US Females Who Died From 1969 to 2020 by State **eTable 2.** Years to Double the Death Rate After Age 35 by State and Cohort in US Males Who Died From 1969 to 2020 by State **eFigure 1.** Range of Ages and Years With Available Data (Solid Fill) and Estimated Mortality Used in the Summaries (Hashed Fill) **eFigure 2.** Observed (Dots) and Fitted (Lines) Death Rates for Females by Cohort **eFigure 3.** Yearly Estimates of the Female Mortality Rate per 100 000 by Cohort for Washington, DC (DC), New York (NY), and Oklahoma (OK) **eFigure 4.** Period Life Expectancy at 40 Years of Age for US Females by State, Region, and Calendar Year (1969, 1995, and 2020) **eFigure 5.** Period Life Expectancy at 40 Years of Age for US Males by State and Calendar Year (1969, 1995, and 2020) **eFigure 6.** Cohort Life Expectancy at 40 Years of Age for US Females by State and Birth Cohort (1900, 1950, and 2000) **eFigure 7.** Cohort Life Expectancy at 40 Years of Age for US Males by State and Birth Cohort (1900, 1950, and 2000)
eReferences

Supplement 2.
Data Sharing Statement
