# Supplementary material for: All-Cause Mortality and Life Expectancy by Birth Cohort Across US States
Source: JAMA Netw Open. 2025 Apr 28;8(4):e257695. doi: 10.1001/jamanetworkopen.2025.7695 (PMC12038512; doi:10.1001/jamanetworkopen.2025.7695)
Supplement: Supplement 1. — eMethods. Data Collection and Analysis eTable 1. Years to Double the Death Rate After Age 40 by State and Cohort in US Females Who Died From 1969 to 2020 by State eTable 2. Years to Double the Death Rate After Age 35 by State and Cohort in US Males Who Died From 1969 to 2020 by State eFigure 1. Range of Ages and Years With Available Data (Solid Fill) and Estimated Mortality Used in the Summaries (Hashed Fill) eFigure 2. Observed (Dots) and Fitted (Lines) Death Rates for Females by Cohort eFigure 3. Yearly Estimates of the Female Mortality Rate per 100 000 by Cohort for Washington, DC (DC), New York (NY), and Oklahoma (OK) eFigure 4. Period Life Expectancy at 40 Years of Age for US Females by State, Region, and Calendar Year (1969, 1995, and 2020) eFigure 5. Period Life Expectancy at 40 Years of Age for US Males by State and Calendar Year (1969, 1995, and 2020) eFigure 6. Cohort Life Expectancy at 40 Years of Age for US Females by State and Birth Cohort (1900, 1950, and 2000) eFigure 7. Cohort Life Expectancy at 40 Years of Age for US Males by State and Birth Cohort (1900, 1950, and 2000) eReferences [file jamanetwopen-e257695-s001.pdf]

Supplementary Online Content

Holford TR, McKay L, Tam J, Jeon J, Meza R. All-cause mortality and life expectancy by birth cohort across US states. *JAMA Netw Open.* 2025;8(4):e257695. doi:10.1001/jamanetworkopen.2025.7695

eMethods. Data Collection and Analysis

eTable 1. Years to Double the Death Rate After Age 40 by State and Cohort in US Females Who Died From 1969 to 2020 by State

eTable 2. Years to Double the Death Rate After Age 35 by State and Cohort in US Males Who Died From 1969 to 2020 by State

eFigure 1. Range of Ages and Years With Available Data (Solid Fill) and Estimated Mortality Used in the Summaries (Hashed Fill)

eFigure 2. Observed (Dots) and Fitted (Lines) Death Rates for Females by Cohort

eFigure 3. Yearly Estimates of the Female Mortality Rate per 100 000 by Cohort for Washington, DC (DC), New York (NY), and Oklahoma (OK)

eFigure 4. Period Life Expectancy at 40 Years of Age for US Females by State, Region, and Calendar Year (1969, 1995, and 2020)

eFigure 5. Period Life Expectancy at 40 Years of Age for US Males by State and Calendar Year (1969, 1995, and 2020)

eFigure 6. Cohort Life Expectancy at 40 Years of Age for US Females by State and Birth Cohort (1900, 1950, and 2000)

eFigure 7. Cohort Life Expectancy at 40 Years of Age for US Males by State and Birth Cohort (1900, 1950, and 2000)

eReferences.

This supplementary material has been provided by the authors to give readers additional information about their work.

© 2025 Holford TR et al. *JAMA Network Open*.

eMethods. Data Collection and Analysis

Data used in this analysis are available for ages 0-84 in the years 1969-2020. eFigure 1 provides a graphical display of the range of ages and calendar years for which data are available. In the analysis, an age-period-cohort model is fitted, and the resulting estimates of mortality rates for the ages and the cohorts extend beyond the range covered by the rectangle representing and age-period range of available data. eFigure 1 also shows a parallelogram representing the ages and cohorts with estimates from the model fitting. Estimates are obtained for ages older than 84, which are needed to estimate life expectancy. In addition, the full range of ages are not represented in any cohort, but some data are available for each cohort included in the analysis. For the earliest cohorts, data are only available for the older ages, and later cohorts only have data from the younger ages.

Age-specific patterns in human mortality change over a lifetime, from a relatively high rate in infancy, followed by a decline to their lowest levels before age 10. Rates subsequently increase, affected by risky behaviors during adolescence and young adulthood before settling into the underlying biology of aging that drives mortality rates higher for the remaining years of life. Gompertz,[1] in 1825, noted that the increase in mortality rates by age in adults is characterized quantitatively by a linear increase in the log rate (i.e., exponential growth), which still holds true today among individuals over age 30. This linear relationship between log mortality and age is seen across human populations [2] and has been observed in other species.[3] Taken together, this knowledge facilitates the comparison of patterns of mortality between different state populations.

To illustrate the fit of the model to these data, a model that includes the Gompertz assumption of linear trend for the log rate with age after age 35, eFigure 2 shows the observed (dots) and fitted (lines) death rates among California females. The general mortality pattern shows expected trends of declining mortality after infancy followed by gradually rising mortality over the life course. The bump that appears in the late teens and early twenties is thought to be the result of risky behavior at this time of life. This figure only shows results for females in California. Similar displays were constructed for males, and for

© 2025 Holford TR et al. *JAMA Network Open*.

each state. However, the number of deaths in some states are less than ten, and confidentiality concerns forbid the general release of these results.

eFigure 3 shows female mortality data for vicennial cohorts in three areas: DC, New York (NY), and Oklahoma (OK) (see https://mortality.cisnetsmokingparameters.org/states/ for mortality rates for all states). Successive cohorts for New York experienced steadily lower mortality over the twentieth century. Mortality in Oklahoma changed little from 1900 to 2000 cohorts, which is easiest to see by following the lowest part of the Oklahoma curves (dotted lines) for successive cohorts. For the 1900 cohort, Oklahoma had lower mortality than New York, but for the most recent cohorts, it was considerably higher. Oklahoma's linear trend line after age 35 was also steeper, indicating a faster effect of aging in older ages. Rates for DC declined steadily from 1900 to 2000, but it had a shallower linear slope after age 35 compared to other states.

The calculation of life expectancy is based on death rate estimates for ages 0-119. eFigures 4 and 5 show period life expectancy estimates for each state at age 40 for females and males, respectively. The patterns among the states are like those seen for life expectancy at birth shown in the main body of text.

Cohort life expectancies at age 40 are shown in eFigures 6 and 7 for females and males, respectively. The spatial patterns among the states are like those seen for life expectancy at birth, which are shown in the text.

Times required to double the mortality rate after age 35 by state, along with 95% confidence limits are shown in eTable 1 for females. Similar results for males are shown in eTable S2. The confidence limits are relatively narrow, suggesting that many of the differences are statistically significant.

© 2025 Holford TR et al. *JAMA Network Open*.

eTable 1. Years to Double the Death Rate After Age 40 by State and Cohort in US Females Who Died From 1969 to 2020 by State

State	Rate Doubling Time	95% C.I.	State	Rate Doubling Time	95% C.I.
District of Columbia	12.27	(12.11 , 12.44)	Alabama	8.50	(8.46 , 8.55)
New York	9.39	(9.34 , 9.44)	South Dakota	8.46	(8.37 , 8.55)
Florida	9.28	(9.22 , 9.34)	Washington	8.45	(8.40 , 8.50)
South Carolina	9.25	(9.19 , 9.31)	Colorado	8.44	(8.39 , 8.50)
Hawaii	9.15	(9.05 , 9.24)	Missouri	8.39	(8.35 , 8.44)
Alaska	9.07	(8.93 , 9.21)	Tennessee	8.39	(8.34 , 8.43)
California	9.05	(9.00 , 9.09)	Ohio	8.39	(8.35 , 8.43)
Georgia	9.02	(8.97 , 9.07)	Oregon	8.29	(8.24 , 8.34)
Arizona	8.94	(8.87 , 9.00)	North Dakota	8.29	(8.20 , 8.38)
Nevada	8.93	(8.85 , 9.01)	West Virginia	8.28	(8.23 , 8.34)
Delaware	8.92	(8.83 , 9.02)	New Hampshire	8.28	(8.21 , 8.35)
Louisiana	8.92	(8.87 , 8.97)	Wyoming	8.25	(8.15 , 8.35)
Maryland	8.87	(8.82 , 8.93)	Vermont	8.24	(8.15 , 8.33)
New Mexico	8.87	(8.80 , 8.95)	Indiana	8.23	(8.19 , 8.27)
New Jersey	8.82	(8.77 , 8.87)	Maine	8.22	(8.16 , 8.28)
Illinois	8.80	(8.76 , 8.85)	Wisconsin	8.19	(8.14 , 8.23)
North Carolina	8.74	(8.69 , 8.79)	Kentucky	8.18	(8.13 , 8.22)
Mississippi	8.68	(8.62 , 8.74)	Arkansas	8.17	(8.12 , 8.22)
Michigan	8.63	(8.59 , 8.67)	Minnesota	8.17	(8.13 , 8.22)
Virginia	8.62	(8.58 , 8.67)	Nebraska	8.14	(8.08 , 8.19)
Pennsylvania	8.60	(8.56 , 8.65)	Utah	8.05	(7.98 , 8.12)
Massachusetts	8.60	(8.55 , 8.65)	Idaho	8.05	(7.97 , 8.12)
Connecticut	8.59	(8.54 , 8.65)	Iowa	7.98	(7.93 , 8.03)
Texas	8.53	(8.48 , 8.57)	Kansas	7.98	(7.93 , 8.03)
Rhode Island	8.52	(8.45 , 8.59)	Oklahoma	7.96	(7.92 , 8.01)
Montana	8.50	(8.42 , 8.59)	**United States**	**8.72**	**(8.68 , 8.76)**

Geographic Divisions:

- Northeast
  - New England
  - Middle Atlantic
- Midwest
  - East North Central
  - West North Central
- West
  - Mountain
  - Pacific
- South
  - South Atlantic
  - East South Central
  - West South Central

© 2025 Holford TR et al. *JAMA Network Open*.

eTable 2. Years to Double the Death Rate After Age 35 by State and Cohort in US Males Who Died From 1969 to 2020 by State

State	Rate Doubling Time	95% C.I.	State	Rate Doubling Time	95% C.I.
District of Columbia	15.64	(15.37 , 15.92)	Hawaii	9.84	(9.75 , 9.93)
Florida	11.47	(11.39 , 11.55)	Michigan	9.83	(9.78 , 9.89)
New York	11.07	(10.99 , 11.15)	West Virginia	9.83	(9.76 , 9.89)
South Carolina	11.06	(10.99 , 11.14)	Connecticut	9.77	(9.71 , 9.84)
Alaska	11.02	(10.86 , 11.19)	Missouri	9.76	(9.70 , 9.82)
Arizona	11.02	(10.93 , 11.10)	Arkansas	9.75	(9.69 , 9.82)
New Mexico	10.97	(10.86 , 11.08)	Vermont	9.70	(9.59 , 9.81)
Georgia	10.74	(10.67 , 10.81)	Utah	9.67	(9.58 , 9.75)
North Carolina	10.62	(10.55 , 10.68)	Washington	9.64	(9.58 , 9.70)
Louisiana	10.55	(10.48 , 10.62)	Kentucky	9.62	(9.57 , 9.68)
California	10.52	(10.46 , 10.59)	Rhode Island	9.61	(9.53 , 9.69)
Nevada	10.46	(10.37 , 10.56)	Oregon	9.60	(9.54 , 9.66)
Delaware	10.37	(10.27 , 10.47)	South Dakota	9.58	(9.49 , 9.68)
Maryland	10.34	(10.27 , 10.41)	Maine	9.51	(9.43 , 9.58)
Mississippi	10.18	(10.11 , 10.25)	Ohio	9.42	(9.37 , 9.47)
Texas	10.17	(10.11 , 10.23)	New Hampshire	9.40	(9.32 , 9.49)
Illinois	10.15	(10.09 , 10.22)	Oklahoma	9.39	(9.34 , 9.45)
Alabama	10.13	(10.06 , 10.19)	Idaho	9.38	(9.29 , 9.47)
New Jersey	10.08	(10.01 , 10.15)	Indiana	9.30	(9.25 , 9.35)
Virginia	10.06	(10.00 , 10.12)	North Dakota	9.26	(9.16 , 9.35)
Wyoming	10.03	(9.90 , 10.16)	Minnesota	9.22	(9.17 , 9.28)
Tennessee	9.94	(9.89 , 10.00)	Nebraska	9.15	(9.09 , 9.22)
Colorado	9.90	(9.82 , 9.97)	Wisconsin	9.13	(9.08 , 9.18)
Massachusetts	9.90	(9.84 , 9.96)	Kansas	9.09	(9.03 , 9.15)
Montana	9.89	(9.79 , 9.99)	Iowa	8.95	(8.90 , 9.01)
Pennsylvania	9.89	(9.83 , 9.94)	**United States**	**10.18**	**(10.13 , 10.24)**

Geographic Divisions:

- Northeast
  - New England
  - Middle Atlantic
- Midwest
  - East North Central
  - West North Central
- West
  - Mountain
  - Pacific
- South
  - South Atlantic
  - East South Central
  - West South Central

© 2025 Holford TR et al. *JAMA Network Open*.

eFigure 1. Range of Ages and Years With Available Data (Solid Fill) and Estimated Mortality Used in the Summaries (Hashed Fill)

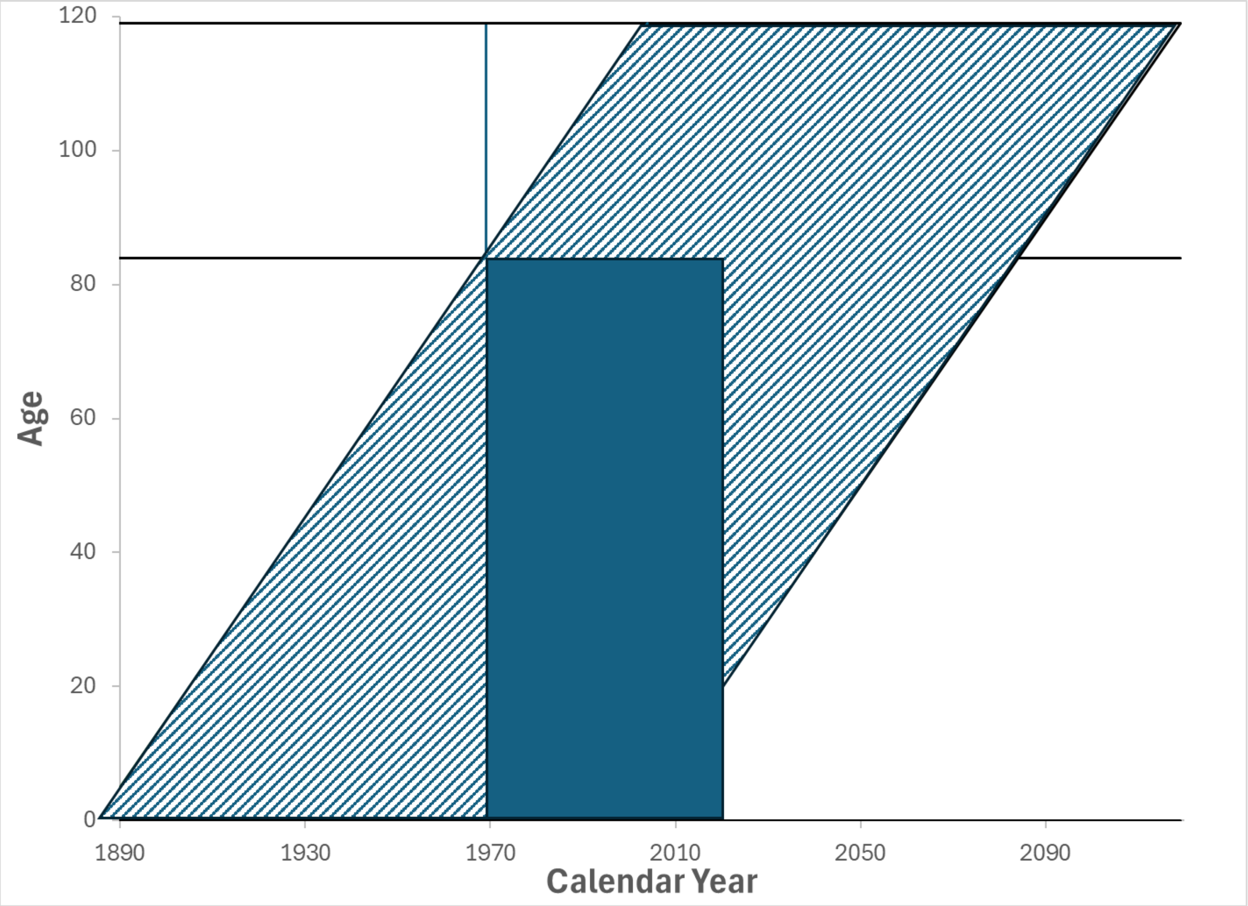

© 2025 Holford TR et al. *JAMA Network Open*.

eFigure 2. Observed (Dots) and Fitted (Lines) Death Rates for Females by Cohort

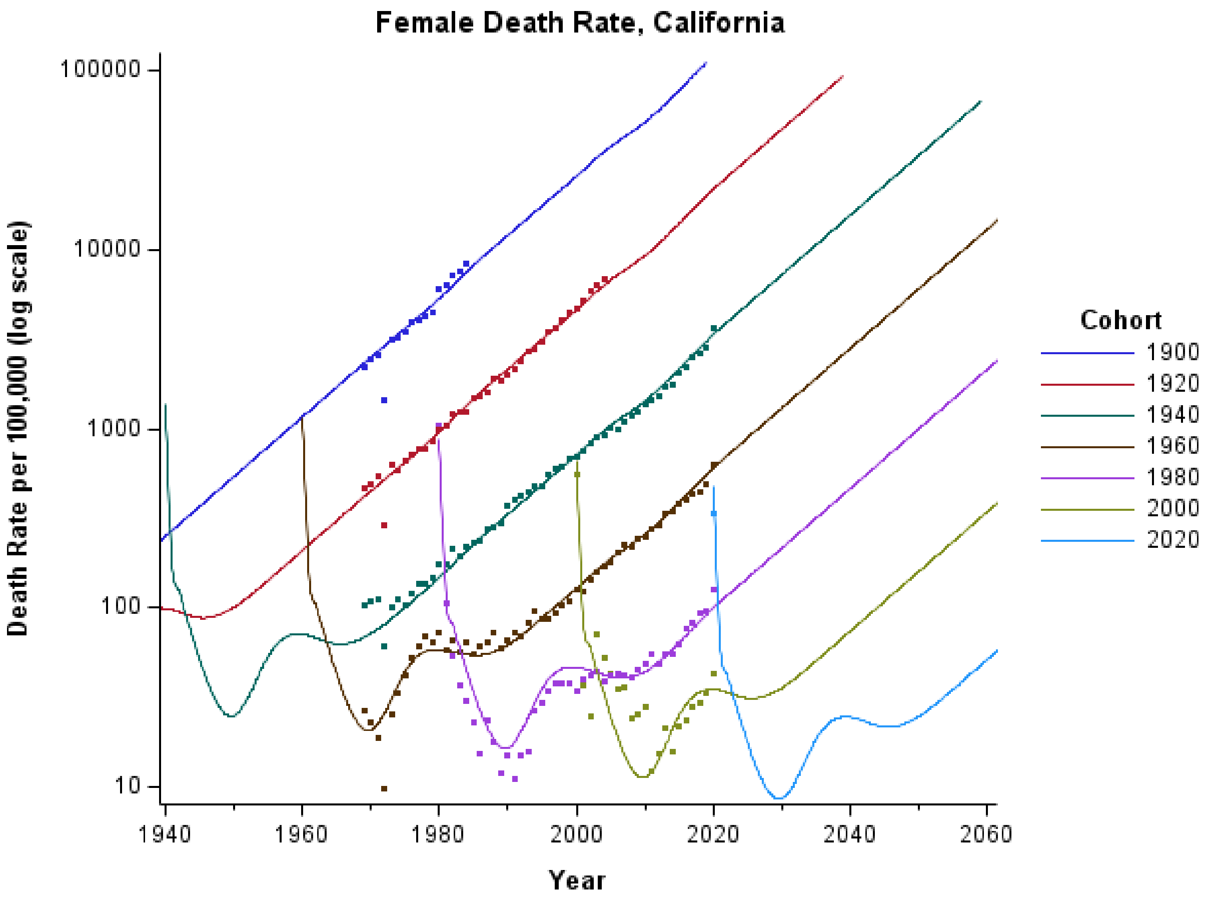

© 2025 Holford TR et al. *JAMA Network Open*.

eFigure 3. Yearly Estimates of the Female Mortality Rate per 100 000 by Cohort for Washington, DC (DC), New York (NY), and Oklahoma (OK)

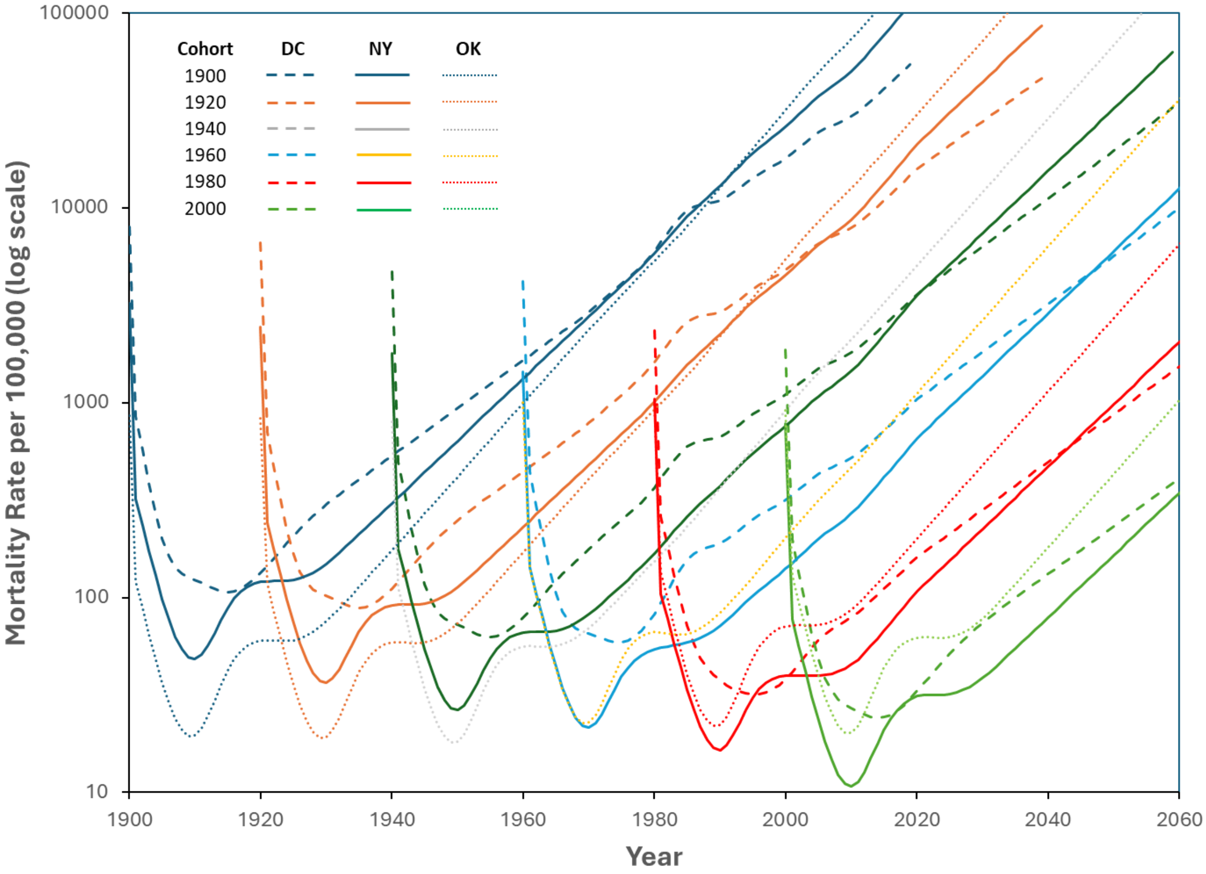

© 2025 Holford TR et al. *JAMA Network Open*.

eFigure 4. Period Life Expectancy at 40 Years of Age for US Females by State, Region, and Calendar Year (1969, 1995, and 2020)

State	1969	1995	2020
Hawaii	39.1	43.2	45.9
California	37.8	40.7	44.0
Florida	38.5	41.7	43.5
Connecticut	37.8	40.9	43.4
New York	36.5	39.9	43.4
Massachusetts	37.3	40.8	43.1
Colorado	38.2	41.1	43.1
Minnesota	38.7	41.7	43.0
Washington	38.0	40.9	42.8
New Jersey	36.4	39.9	42.7
Arizona	38.1	41.4	42.7
Vermont	37.5	40.7	42.6
Rhode Island	37.3	41.0	42.5
New Hampshire	36.9	40.7	42.4
Oregon	38.4	40.4	42.4
Utah	38.1	41.6	42.1
North Dakota	38.9	42.2	42.1
Wisconsin	37.9	41.0	42.1
Delaware	36.3	39.6	42.1
Idaho	38.2	41.2	42.0
Maryland	36.4	39.5	41.9
District of Columbia	35.3	37.6	41.9
South Dakota	38.8	41.9	41.8
Virginia	36.7	39.7	41.8
Illinois	36.4	39.7	41.8
Nebraska	38.8	41.4	41.8

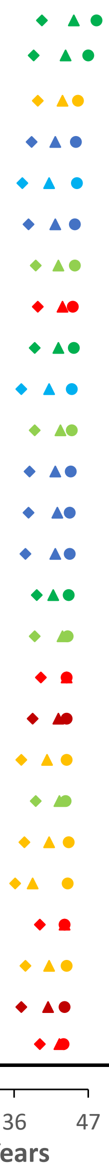

State	1969	1995	2020
Maine	36.9	40.3	41.7
Alaska	35.7	40.5	41.6
Montana	37.9	41.0	41.6
Iowa	38.5	41.5	41.6
Pennsylvania	36.1	39.8	41.4
Texas	37.5	40.1	41.3
New Mexico	37.0	41.3	41.2
Nevada	36.4	39.3	41.2
Wyoming	37.7	40.6	41.1
North Carolina	36.4	39.8	41.0
Michigan	36.7	39.7	40.8
Kansas	38.6	41.0	40.7
Georgia	36.0	39.1	40.5
South Carolina	35.4	39.1	40.4
Missouri	37.1	39.7	40.1
Ohio	36.6	39.5	39.9
Indiana	36.8	39.5	39.7
Louisiana	35.7	38.5	39.1
Tennessee	36.7	39.1	38.9
Arkansas	37.5	39.5	38.8
Oklahoma	38.1	39.4	38.7
Alabama	36.3	39.0	38.6
Kentucky	36.6	39.1	38.4
West Virginia	36.1	38.8	38.1
Mississippi	35.9	38.7	38.1
**United States**	**37.0**	**40.2**	**41.7**

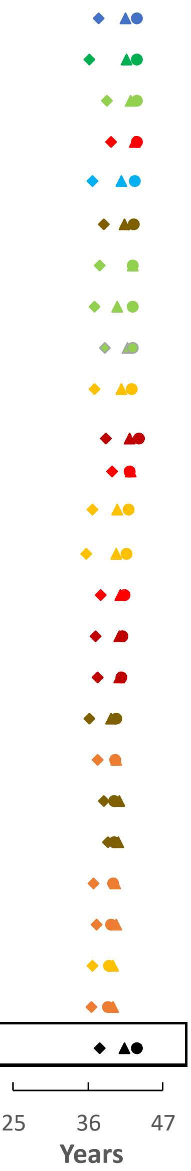

Year
◆ 1969 ▲ 1995 ■ 2020

Geographic Divisions:

Northeast
- New England
- Middle Atlantic

Midwest
- East North Central
- West North Central

West
- Mountain
- Pacific

South
- South Atlantic
- East South Central
- West South Central

***Northeast**

New England: Connecticut, Maine, Massachusetts, New Hampshire, Rhode Island, and Vermont
Middle Atlantic: New Jersey, New York, and Pennsylvania

© 2025 Holford TR et al. *JAMA Network Open*.

Midwest
East North Central: Illinois, Indiana, Michigan, Ohio, and Wisconsin
West North Central: Iowa, Kansas, Minnesota, Missouri, Nebraska, North Dakota, and South Dakota
South
South Atlantic: Delaware, District of Columbia, Florida, Georgia, Maryland, North Carolina, South Carolina, Virginia, and West Virginia
East South Central: Alabama, Kentucky, Mississippi, and Tennessee
West South Central: Arkansas, Louisiana, Oklahoma, and Texas
West
Mountain: Arizona, Colorado, Idaho, Montana, Nevada, New Mexico, Utah, and Wyoming
Pacific: Alaska, California, Hawaii, Oregon, and Washington. [4]

© 2025 Holford TR et al. *JAMA Network Open*.

eFigure 5. Period Life Expectancy at 40 Years of Age for US Males by State and Calendar Year (1969, 1995, and 2020)

State	1969	1995	2020
Hawaii	34.1	37.4	39.7
California	31.6	35.4	39.2
Minnesota	32.3	36.4	39.0
Utah	32.3	37.2	39.0
Colorado	31.9	36.2	38.9
Washington	31.3	35.9	38.7
Connecticut	31.7	35.7	38.7
New York	30.6	34.3	38.5
Massachusetts	30.7	35.4	38.4
Florida	31.5	35.7	38.4
Vermont	30.6	35.5	38.4
Oregon	31.8	35.6	38.3
Idaho	31.9	36.3	38.2
New Hampshire	30.2	35.8	38.1
Alaska	30.6	35.7	37.9
New Jersey	30.6	34.7	37.9
Arizona	31.1	35.8	37.8
Rhode Island	31.1	35.4	37.7
Wisconsin	31.9	35.8	37.7
Virginia	29.7	34.3	37.6
Nebraska	32.2	35.7	37.5
North Dakota	32.3	36.2	37.5
Wyoming	30.7	35.6	37.4
Montana	31.0	35.6	37.4
Maryland	29.8	34.0	37.2
Maine	30.2	35.2	37.1

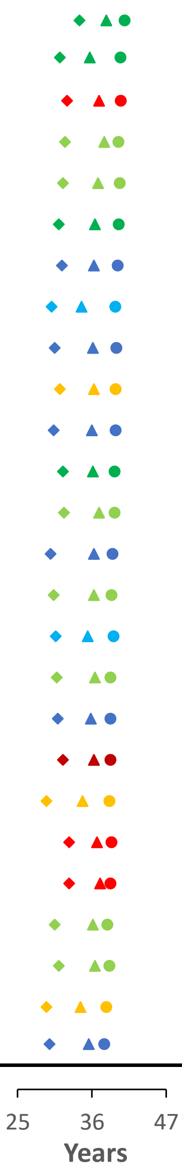

State	1969	1995	2020
South Dakota	32.0	35.6	37.1
Iowa	31.9	35.9	37.1
Delaware	29.4	34.4	37.1
Illinois	30.1	34.2	37.1
Pennsylvania	30.0	34.4	36.7
Texas	30.9	34.3	36.7
Nevada	30.2	33.8	36.6
Kansas	32.0	35.5	36.5
North Carolina	29.1	33.6	36.5
Michigan	30.4	34.5	36.5
District of Columbia	27.6	28.7	36.1
Georgia	28.5	33.0	36.0
New Mexico	31.0	36.0	36.0
South Carolina	28.0	33.0	35.4
Missouri	30.5	34.1	35.4
Ohio	30.4	34.3	35.3
Indiana	30.4	34.3	35.3
Arkansas	30.7	33.4	34.3
Oklahoma	31.1	33.7	34.3
Tennessee	30.1	33.2	34.2
Louisiana	29.3	32.7	34.2
Kentucky	29.9	33.3	33.8
Alabama	29.3	32.9	33.8
West Virginia	29.4	33.5	33.6
Mississippi	29.2	32.1	32.8
**United States**	**30.6**	**34.6**	**37.1**

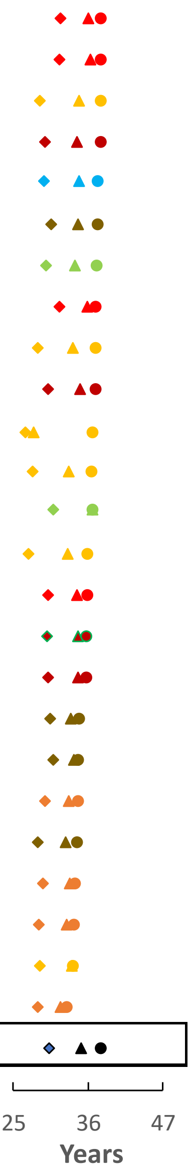

Year
◆ 1969 ▲ 1995 ■ 2020

Geographic Divisions:

Northeast
- New England
- Middle Atlantic

Midwest
- East North Central
- West North Central

West
- Mountain
- Pacific

South
- South Atlantic
- East South Central
- West South Central

© 2025 Holford TR et al. *JAMA Network Open*.

eFigure 6. Cohort Life Expectancy at 40 Years of Age for US Females by State and Birth Cohort (1900, 1950, and 2000)

State	1900	1950	2000
District of Columbia	34.7	43.5	56.6
New York	36.5	45.1	53.6
California	37.9	45.8	52.8
Hawaii	39.5	47.8	50.5
Massachusetts	37.7	45.5	50.2
New Jersey	36.5	44.5	49.8
Washington	38.4	44.8	49.3
Connecticut	38.0	45.6	48.7
Illinois	36.8	43.3	48.1
Minnesota	39.3	45.0	48.0
Oregon	38.8	44.2	47.8
Florida	38.9	45.2	47.4
Rhode Island	37.7	44.4	47.2
New Hampshire	37.5	45.0	46.7
Vermont	37.9	45.2	46.6
Colorado	38.7	45.4	46.6
Nevada	36.6	42.9	46.6
Texas	38.0	42.6	46.6
Virginia	37.0	43.8	46.3
Arizona	38.7	44.7	45.9
Maine	37.6	44.0	45.9
Georgia	36.3	42.1	45.5
Iowa	39.3	43.1	45.3
Maryland	36.5	43.9	45.1
Nebraska	39.4	43.5	44.9
South Carolina	35.7	42.5	44.6

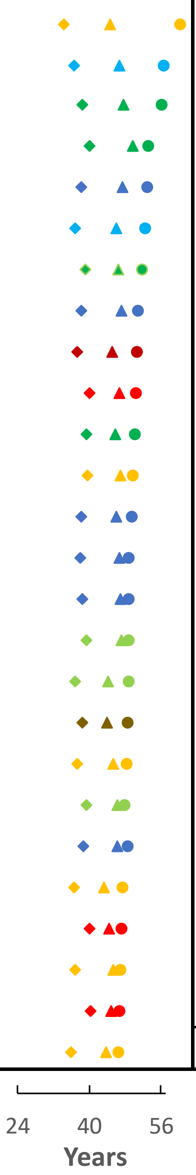

State	1900	1950	2000
North Carolina	37.0	42.7	44.6
Idaho	39.1	44.0	44.4
Pennsylvania	36.4	43.2	44.2
Utah	38.7	44.2	44.2
Wisconsin	38.4	43.8	43.8
Michigan	37.1	42.3	43.8
Alaska	36.1	45.1	43.8
Wyoming	38.5	43.0	43.7
Montana	38.5	44.3	43.6
New Mexico	37.9	43.7	43.0
Delaware	36.5	44.3	43.0
South Dakota	39.6	44.0	42.9
North Dakota	39.5	44.1	42.2
Kansas	39.4	42.1	41.8
Missouri	37.7	41.6	41.6
Ohio	37.0	41.6	40.7
Indiana	37.3	41.4	40.4
Louisiana	35.9	40.6	40.3
Tennessee	37.2	40.5	39.3
Mississippi	36.7	39.4	39.2
Alabama	36.9	40.0	39.2
Arkansas	38.1	40.0	38.8
Kentucky	37.3	40.0	38.5
Oklahoma	38.8	39.5	38.1
West Virginia	36.9	40.3	37.5
**United States**	**37.4**	**43.4**	**45.9**

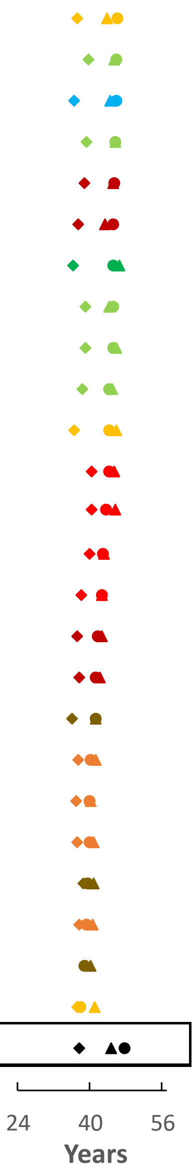

Year
◆ 1900 ▲ 1950 ■ 2000

Geographic Divisions:

Northeast
- New England
- Middle Atlantic

Midwest
- East North Central
- West North Central

West
- Mountain
- Pacific

South
- South Atlantic
- East South Central
- West South Central

© 2025 Holford TR et al. *JAMA Network Open*.

eFigure 7. Cohort Life Expectancy at 40 Years of Age for US Males by State and Birth Cohort (1900, 1950, and 2000)

State	1900	1950	2000
District of Columbia	25.3	35.4	52.0
New York	29.0	39.2	50.4
California	30.4	40.0	49.3
Massachusetts	29.8	40.4	46.9
Washington	30.6	40.2	46.7
Oregon	31.1	39.5	46.6
Florida	30.6	39.3	45.9
Vermont	29.8	41.0	45.8
Minnesota	31.8	40.7	45.7
New Jersey	29.5	39.2	45.6
Alaska	29.5	40.6	45.4
Connecticut	30.7	40.6	45.1
Texas	30.2	37.7	45.0
Nevada	29.1	37.3	44.8
New Hampshire	29.6	40.6	44.5
Idaho	31.5	40.0	44.3
Arizona	30.2	39.3	44.0
Virginia	29.0	39.3	44.0
Utah	31.6	41.1	44.0
Illinois	29.2	38.4	43.8
Colorado	31.0	41.0	43.8
Hawaii	33.8	40.9	43.8
Rhode Island	30.2	39.3	43.7
Nebraska	31.7	39.1	43.3
Montana	30.4	39.7	43.1
Georgia	27.9	36.9	43.0

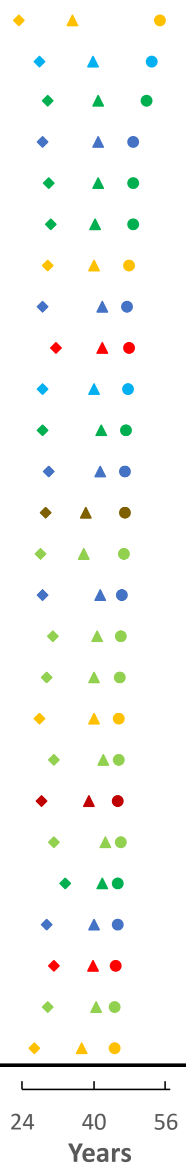

State	1900	1950	2000
Iowa	31.5	38.6	43.0
Wyoming	30.0	39.7	43.0
North Carolina	28.6	37.9	42.8
Maine	29.7	39.4	42.1
New Mexico	30.3	38.6	41.8
Wisconsin	31.3	39.5	41.5
Michigan	29.6	37.7	41.2
South Carolina	27.5	36.8	40.8
Maryland	28.6	38.8	40.6
North Dakota	31.8	39.6	40.6
Pennsylvania	29.1	38.3	40.4
South Dakota	31.5	39.2	40.1
Kansas	31.7	37.9	39.4
Delaware	28.3	39.1	39.0
Missouri	29.9	37.0	38.5
Indiana	29.9	36.9	37.5
Ohio	29.8	37.0	37.5
Arkansas	30.4	35.3	37.0
Louisiana	28.5	35.6	37.0
Tennessee	29.6	35.7	37.0
Oklahoma	30.9	35.3	36.8
Kentucky	29.7	35.7	36.6
Alabama	29.0	34.9	36.5
Mississippi	29.0	34.1	36.0
West Virginia	29.2	35.9	35.9
**United States**	**29.8**	**38.4**	**43.2**

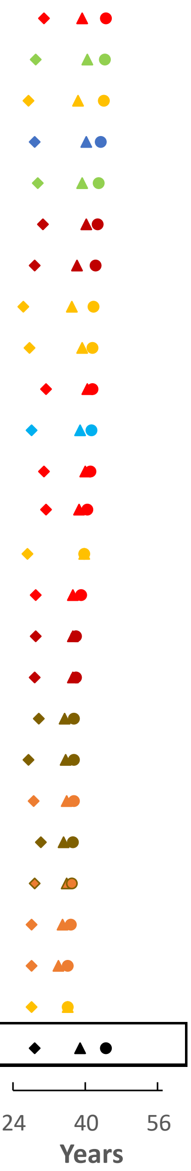

Year
◆ 1900 ▲ 1950 ■ 2000

Geographic Divisions:

Northeast
- New England
- Middle Atlantic

Midwest
- East North Central
- West North Central

West
- Mountain
- Pacific

South
- South Atlantic
- East South Central
- West South Central

© 2025 Holford TR et al. *JAMA Network Open*.

eReferences.

1. Gompertz B. On the nature of the function expressive of the law of human mortality, and on a new mode of determining the value of life contingencies. *Philosophical Transactions of the Royal Society of London*. 1825;115:513-583.
2. Meza R, Pourbohloul B, Brunham RC. Birth cohort patterns suggest that infant survival predicts adult mortality rates. *J Dev Orig Health Dis*. 2010;1(3):174-183. doi:10.1017/S2040174410000218
3. Sacher GA. On the statistical nature of mortality, with especial reference to chronic radiation mortality. *Radiology*. 1956;67(2):250-257. doi:10.1148/67.2.250
4. Centers for Disease Control and Prevention. Geographic division or region. National Center for Health Statistics. Accessed January 29, 2025, 2025. https://www.cdc.gov/nchs/hus/sources-definitions/geographic-region.htm#:~:text=The%20nine%20divisions%20are%3A,%2C%20Michigan%2C%20Ohio%2C%20and%20Wisconsin

© 2025 Holford TR et al. *JAMA Network Open*.
