# Supplementary material for: All-Cause Mortality and Life Expectancy by Birth Cohort Across US States
Source: JAMA Netw Open. 2025 Apr 28;8(4):e257695. doi: 10.1001/jamanetworkopen.2025.7695 (PMC12038512; doi:10.1001/jamanetworkopen.2025.7695)
Supplement: Supplement 2. — Data Sharing Statement [file jamanetwopen-e257695-s002.pdf]

Data Sharing Statement

Holford. All-Cause Mortality and Life Expectancy by Birth Cohort Across US States. *JAMA Netw Open*. Published April 28, 2025. doi:10.1001/jamanetworkopen.2025.7695

Data
Data available: No

Additional Information
Explanation for why data not available: The data used in this study are from federal data bases that are publicly available.
